# Pressure-Induced Enhancement of Superconductivity in 3R-TaSe_2_ Single Crystals

**DOI:** 10.3390/ma19214584

**Published:** 2026-10-29

**Authors:** Jiahao Wang, Wei Wu, Shilin Zhu, Yanmin Zhang, Lei Qin, Jianlin Luo

**Affiliations:** 1 Beijing Key Laboratory for Sensor, Beijing Information Science and Technology University, Beijing 100192, China; 1201002026@cnu.edu.cn (J.W.);; 2 School of Applied Science, Beijing Information Science and Technology University, Beijing 100192, China; 3 Beijing National Laboratory for Condensed Matter Physics, Institute of Physics, Chinese Academy of Sciences, Beijing 100190, China

**Keywords:** 3R-TaSe_2_, pressure, superconductivity, charge-density-wave (CDW), anisotropic upper critical field

## Abstract

Layered transition-metal dichalcogenides provide an ideal platform to explore the competition between charge-density-wave (CDW) order and superconductivity. The 3R-stacked TaSe_2_ hosts intrinsic CDW and superconductivity without chemical doping, yet systematic high-pressure experimental evidence remains lacking. Here, we report on systematic studies of pressure phase diagrams of up to 2 GPa in high-quality single crystals of 3R-TaSe_2_. At ambient pressure, a CDW transition emerges near 100 K and bulk superconductivity onset occurs at *T*_c_ ≈ 2.8 K. Upon hydrostatic compression, the CDW transition temperature is monotonically suppressed, while *T*_c_ increases to ~3.1 K at 2 GPa, clearly revealing an antagonistic relationship between these two quantum phases. Hall and thermopower measurements suggest CDW-driven Fermi-surface reconstruction and single-band carrier behavior. From pressure-dependent transport, we construct the *P*(*T*) phase diagram, and anisotropic upper-critical-field data demonstrate quasi-two-dimensional superconductivity in bulk 3R-TaSe_2_. This work offers key experimental insights into competing correlated states in van der Waals layered materials.

## 1. Introduction

Chemical doping, intercalation, electrostatic gating, strain, and hydrostatic pressure are common methods for tuning the electronic structure of transition-metal dichalcogenides (TMDs) [1,2,3,4,5]. These layered materials consist of covalently bonded X-M-X trilayers, which are stacked via weak van der Waals interactions. The subtle interplay between intralayer electronic states and interlayer coupling underpins a rich variety of exotic quantum phenomena, including charge-density-wave (CDW) order, superconductivity, Mott insulating behavior, and even topological electronic states [6,7,8,9]. Chemical doping introduces impurity scattering centers and compromises lattice integrity. Electrostatic gating modulates only the electronic states of a thin surface layer and is inherently incapable of inducing continuous evolution of bulk properties [10]. In contrast, hydrostatic pressure uniformly modulates the interlayer spacing, thereby continuously tuning the strength of interlayer orbital hybridization and electron–phonon coupling without introducing extrinsic defects. As such, pressure serves as a clean, reversible, and quantitatively controllable tuning parameter, ideally suited for systematically probing the interplay between CDW order and superconductivity [11,12,13].

As a prototypical layered transition-metal dichalcogenide, TaSe_2_ exhibits multiple polymorphs, including 1T, 2H, and 3R. The structural and electronic distinctions arise from differences in the coordination geometry of Ta atoms (octahedral versus trigonal prismatic) and the interlayer stacking sequence (AA, AB, or ABC) [14]. These structural variations give rise to markedly different CDW superlattice structures, Fermi-surface topologies, and superconducting pairing mechanisms. In 1T-TaSe_2_, Ta atoms adopt octahedral coordination and stack in an AA fashion. Each unit cell comprises a single Se-Ta-Se trilayer [14]. Strong interlayer coupling stabilizes a high-temperature, commensurate CDW characterized by star-of-David motifs [14]. However, bulk superconductivity is absent under ambient pressure. Superconductivity can be induced only via external perturbations such as hydrostatic pressure or chemical doping [15]. 2H-TaSe_2_ crystallizes in hexagonal symmetry with trigonal-prismatic Ta coordination and AB stacking, whose unit cell encloses two Se-Ta-Se layers [14]. It undergoes successive incommensurate-to-commensurate CDW transitions and exhibits weak intrinsic superconductivity at ambient pressure [16]. Its Fermi-surface nesting is dominated by one main carrier type without double sign reversal in transport measurements. 3R-TaSe_2_ is a rhombohedral polymorph with trigonal-prismatic Ta coordination and ABC-type three-layer stacking. Its unit cell contains three Se-Ta-Se trilayers, leading to lowered crystal symmetry and split Fermi surfaces with coexisting electron and hole pockets, which gives rise to multiband transport behaviors. However, research on 3R-TaSe_2_ remains notably deficient: early work mainly addressed the single-crystal structure and ambient-pressure basic transport [16], while existing high-pressure experiments, despite establishing pressure as a clean tuning knob for competing CDW and superconductivity [17], covered only isolated pressure points, lacking systematic multiband verification and anisotropic critical field data. There is a lack of quantitative evolution relationships between *T_CDW_* and *T_c_* over a pressure range, and multi-dimensional thermodynamic evidence, anisotropic critical fields, and multiband carrier signatures are missing. To date, a *P*(*T*) phase diagram has not been established, preventing a clear quantitative description of the antagonistic strength between CDW and superconductivity.

In this study, we report how hydrostatic pressure can efficiently tune both the CDW order and superconductivity in 3R-TaSe_2_, as revealed by electrical resistivity measurements. High-quality single crystals of 3R-TaSe_2_ were synthesized via the chemical vapor transport (CVT) method, using iodine as the transport agent. Powder X-ray diffraction indicates that the as-grown crystals are predominantly 3R-stacked with good crystalline quality, consistent with previous reports [7]. We carried out comprehensive characterizations including temperature-dependent electrical transport, Hall effect, magnetoresistance and thermopower at ambient pressure. These measurements revealed a CDW transition near 100 K and bulk superconductivity with a critical temperature *T_c_* ≈ 2.8 K. Two orthogonal field orientation upper-critical-field measurements further demonstrated the quasi-two-dimensional nature of superconductivity in bulk 3R-TaSe_2_. Using a piston-cylinder cell, we systematically investigated the pressure-dependent transport properties under hydrostatic pressure up to 2 GPa. With increasing pressure, the CDW transition temperature is monotonically suppressed, whereas the superconducting critical temperature *T_c_* is distinctly enhanced, manifesting the antagonistic competition between CDW order and superconductivity. Based on these pressure-dependent experimental results, we constructed the *P*(*T*) phase diagram for pristine 3R-TaSe_2_.

## 2. Experimental Methods

**Figure 1:**
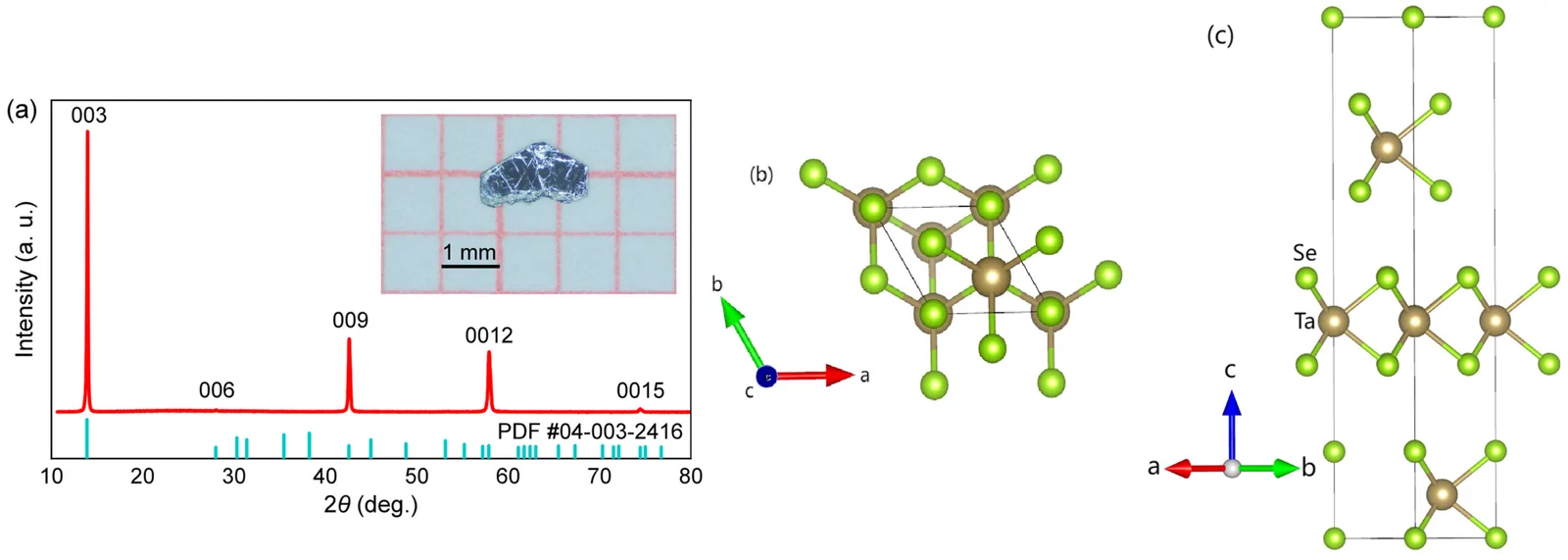
(**a**) X-ray diffraction pattern of a 3R-TaSe_2_ single crystal. The red line and vertical bars represent the measured data and ICDD PDF card results, respectively. The inset shows an optical image of the 3R-TaSe_2_ single crystal. (**b**) Crystal structure of 3R-TaSe_2_ projected onto the *ab* plane. (**c**) The framework of Ta and Se atoms in the (100) plane of 3R-TaSe_2_.

Millimeter-sized single crystals of 3R-TaSe_2_ were synthesized via the chemical vapor transport method in Figure 1. High-purity tantalum powder (99.99%, Alfa Aesar, MA, USA) and selenium powder (99.999%, Alfa Aesar, MA, USA) were sealed in a quartz tube at a molar ratio of 1:2.05, with a slight excess of Se to compensate for high-temperature volatilization. Iodine (99.99%, Alfa Aesar, MA, USA) was employed as the transport agent at a concentration of approximately 5 mg/cm^3^. A temperature gradient of 950 °C to 850 °C was maintained for 14 days, followed by natural cooling, which yielded shiny, flake-like 3R-TaSe_2_ single crystals [7].

All ambient-pressure and low-temperature electrical measurements were carried out on a Quantum Design Physical Property Measurement System (PPMS-9T, Quantum Design, San Diego, CA, USA), covering a temperature range of 2–300 K and a maximum magnetic field of 9 T. The longitudinal resistance *R*(*T*), transverse Hall resistivity *ρ*_xy_(*H*), and magnetoresistance were measured via the standard four-probe technique. Hall resistivity *ρ_xy_*(*H*) was measured under a magnetic field swept from −9 T to 9 T at fixed temperatures. The Hall coefficient *R_H_* was obtained by the linear fitting of *ρ_xy_*(*H*) in the high-field region (5–9 T), where *ρ_xy_* was approximately linear in *H*. The apparent carrier density and mobility were calculated within a single-band approximation: *R_H_* = d*ρ*_xy_/d*H*, *n_app_* = 1/(*e*|*R_H_*|), *μ_app_* = |*R*_H_|/*ρ*_xx_, where *e* was the elementary charge and *ρ*_xx_ was the longitudinal resistivity at zero field. The sign of *R_H_* indicates the dominant carrier type. The uncertainties in *n_app_* and *μ_app_* were propagated from the standard error of the linear fit of *ρ_xy_*(*H*) and the uncertainty in the sample geometry.

We emphasize that the single-band approximation provides only effective parameters. Because the Hall signal changes sign with temperature, indicating multiband transport, two-band fits would be more appropriate. However, due to the limited field range and the complexity of the multiband Fermi surface, we present the single-band results as effective quantities and discuss their temperature evolution qualitatively. The magnetoresistance is defined as

(1) $$\mathrm{MR}(\%)=\frac{\rho (T,H)-\rho (T,0)}{\rho (T,0)}\times 100\%.$$

**Figure 2:**
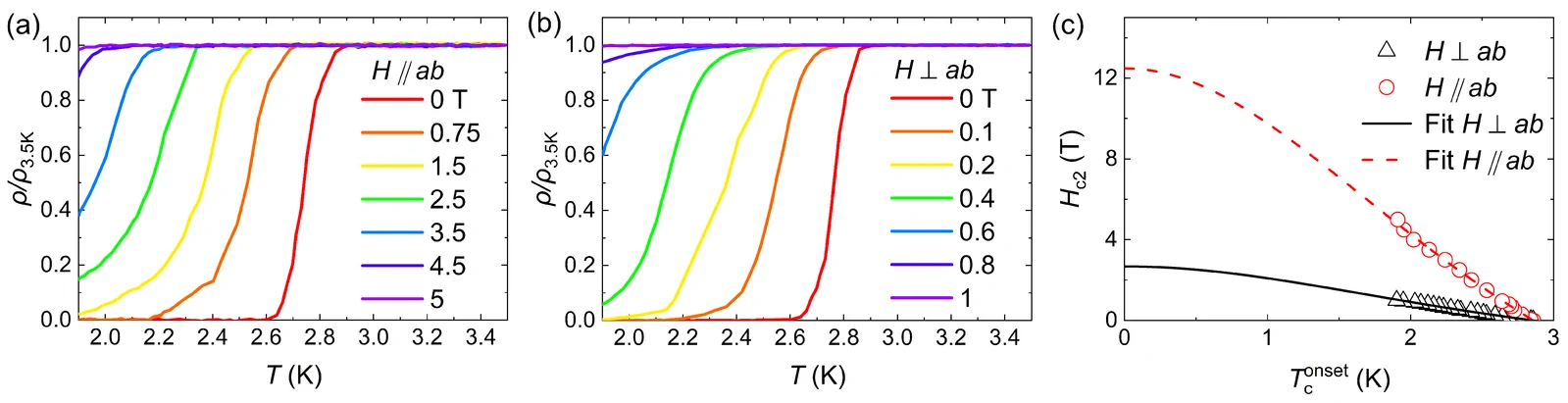
(**a**) Temperature dependence of normalized resistivity *ρ*/*ρ*_3_._5K_ under magnetic fields applied parallel to the *ab* plane (*H*∥*ab*). Curves of different colors correspond to different applied magnetic fields. (**b**) Temperature dependence of normalized resistivity *ρ*/*ρ*_3_._5K_ for magnetic fields perpendicular to the *ab* plane (*H*⊥*ab*). (**c**) *H_c_*_2_ as a function of *T*_c_^onset^ extracted from the *ρ*(*T*, *H*) curves in Figure 2a,b using the onset criterion. Red circles: *H*∥*ab*; black triangles: *H*⊥*ab*. Solid and dashed lines are fits using the anisotropic Ginzburg–Landau model *H_c_*_2_(*T*) = *H_c_*_2_(0)(1−*t*^2^)/(1 + *t*^2^), where *t* = *T*/*T_c_*_0_ and *T_c_*_0_ = 2.858 K. The lines also indicate extrapolation to *T* = 0 K beyond the measured field range.

The superconducting anisotropy was investigated by measuring the upper critical field under magnetic fields applied parallel (*H*∥*ab*) and perpendicular (*H*⊥*ab*) to the crystal *ab*-plane. The upper critical field *H_c_*_2_(*T*) was determined from the onset of the superconducting transition in the *R*(*T*, *H*) curves shown in Figure 2a,b. Here, $T_{c}^{\mathrm{onset}}$(*H*) is defined as the temperature at which the resistance starts to deviate from the normal-state background. For each fixed magnetic field, the onset temperature was extracted, and the corresponding field was plotted against *T_c_*^onset^ in Figure 2c. The *H_c_*_2_(*T*) data were fitted using the anisotropic Ginzburg–Landau model *H_c_*_2_(*T*) = *H_c_*_2_(0)(1−*t*^2^)/(1 + *t*^2^), *t* = *T/T_c_*_0_, where *T_c_*_0_ = 2.858 K was the zero-field onset temperature. The fits were performed separately for *H*∥*ab* and *H*⊥*ab*. High-pressure electrical measurements were performed using a piston-cylinder cell (CTF-HHPC50, C&T Factory, Tokyo, Japan). The cell cylinder is made of nonmagnetic CuBe and NiCrAl alloys with an inner diameter of 5.0 mm and an outer diameter of 25 mm. The standard operating pressure range of this piston-cylinder cell is approximately 2 GPa, which is well within the range of our measurements (0–2.0 GPa). The sample was loaded into a Teflon sample tube together with a piece of high-purity Pb foil, which served as an in situ pressure manometer. Silicone oil was used as the pressure-transmitting medium. Electrical contacts were made using platinum wires attached to the sample with silver epoxy in a standard four-probe configuration. The cell was pressurized at room temperature using a hydraulic press (P-8KG558, RIKEN SEIKI, Ojiya City, Japan) and then clamped. Upon cooling, the silicone oil may solidify, leading to a slightly non-hydrostatic condition at low temperatures. However, the large sample chamber of the piston-cylinder cell (~5 mm diameter) typically maintains a quasi-hydrostatic pressure environment with minimal pressure gradients. The nominal pressure at room temperature was estimated from the load applied by the hydraulic press, though this value may overestimate the actual pressure due to friction between the piston and the Teflon sample tube. Because the pressure can also change upon cooling due to the different thermal expansion coefficients of the cell components, the actual low-temperature pressure was determined in situ using the Pb foil. The known pressure dependence of the Pb superconducting transition temperature *dT_c_*/*dP* was used to convert the measured *T_c_* of Pb into pressure. The pressure uncertainty was estimated to be ±0.05 GPa. Measurements were performed at fixed pressures of 0, 0.6, 1.0, 1.4, and 2.0 GPa.

## 3. Results and Data Analysis

Figure 1a presents the X-ray diffraction (XRD) pattern of the as-grown 3R-TaSe_2_ single crystal, measured using an XRD-7000 diffractometer (Shimadzu Corporation, Kyoto, Japan). The pattern is dominated by the (00*l*) reflections expected for the 3R polytype, including (003), (006), (009), (0012), and (0015), and no other obvious impurity peaks are observed. The XRD results agree with those reported in Ref. [7]. The sharp diffraction profiles indicate good crystalline quality. Optical microscopy shows that the crystals are 1–3 mm in size with smooth, crack-free surfaces. Figure 1b,c illustrate the atomic stacking of 3R-TaSe_2_. In the layered structure, Ta atoms form an in-plane hexagonal sublattice and are symmetrically sandwiched between Se layers. Neighboring atomic slabs are stacked along the c-axis and held together by weak van der Waals forces, which dominate the intrinsic layered nature of 3R-phase TaSe_2_ [14].

**Figure 3:**
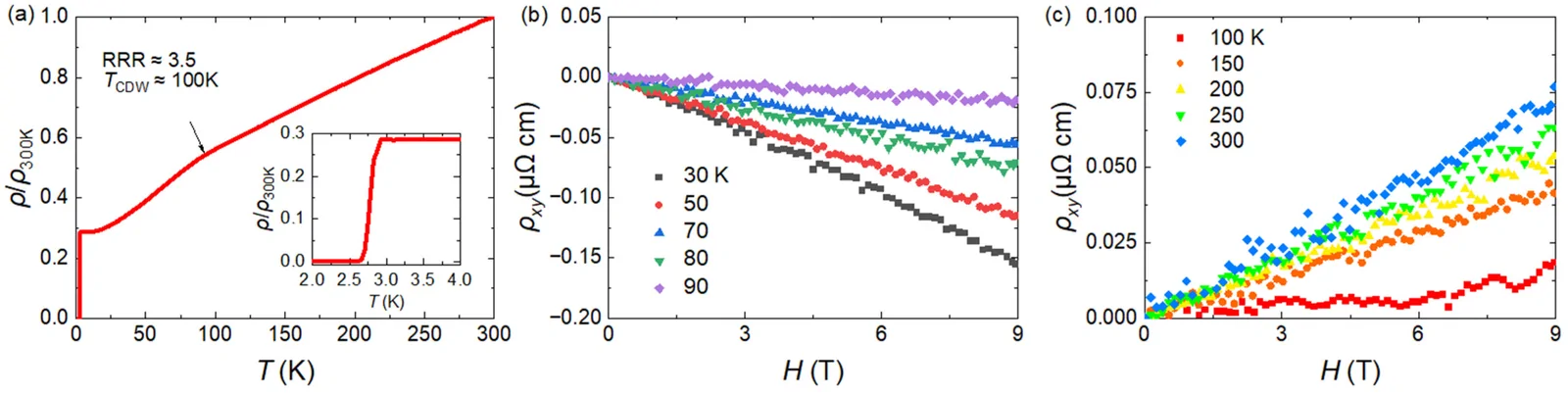
(**a**) Temperature-dependent in-plane normalized electrical resistivity (*ρ*/*ρ*_300K_) of 3R-TaSe_2_. Inset shows the *ρ*/*ρ*_300K_ in the range of 2–4 K, an enlarged view of the superconducting transition with an onset temperature *T*_c_^onset^ and zero temperature *T*_c_^zero^ of 2.93 K and 2.65 K, respectively. (**b**) *H*-dependent Hall resistivity *ρ*_xy_ of 3R-TaSe_2_ at 30–90 K. (**c**) *H*-dependent Hall resistivity *ρ*_xy_ of 3R-TaSe_2_ at 100–300 K.

The ambient-pressure *R*(*T*) curve in Figure 3a exhibits typical metallic behavior from room temperature down to 100 K, with a clear resistance inflection at *T_CDW_* ≈ 100 K, which corresponds to the CDW lattice distortion and Fermi-surface nesting transition [5]. The residual resistivity ratio (RRR), defined as *ρ*_300K_/*ρ*_10K_ with *ρ* denoting resistivity, serves as an indicator for crystalline quality. We obtain RRR ≈ 3.5, consistent with prior reports on 3R-TaSe_2_ single crystals [7]. Upon further cooling to ~2.8 K, the resistance abruptly drops to zero, marking the superconducting transition. The Hall resistivity in Figure 3b,c shows a prominent sign reversal as temperature varies: for *T* > 100 K (CDW-disordered phase), *ρ*_xy_ is positive, indicative of hole-dominated transport; for *T* < 100 K (CDW-ordered phase), *ρ*_xy_ turns negative, revealing electron-dominated transport. This phenomenon arises from Fermi-surface folding induced by the CDW superstructure. Some hole pockets open energy gaps while electron pockets remain intact, which accounts for the carrier-type reversal [18].

**Figure 4:**
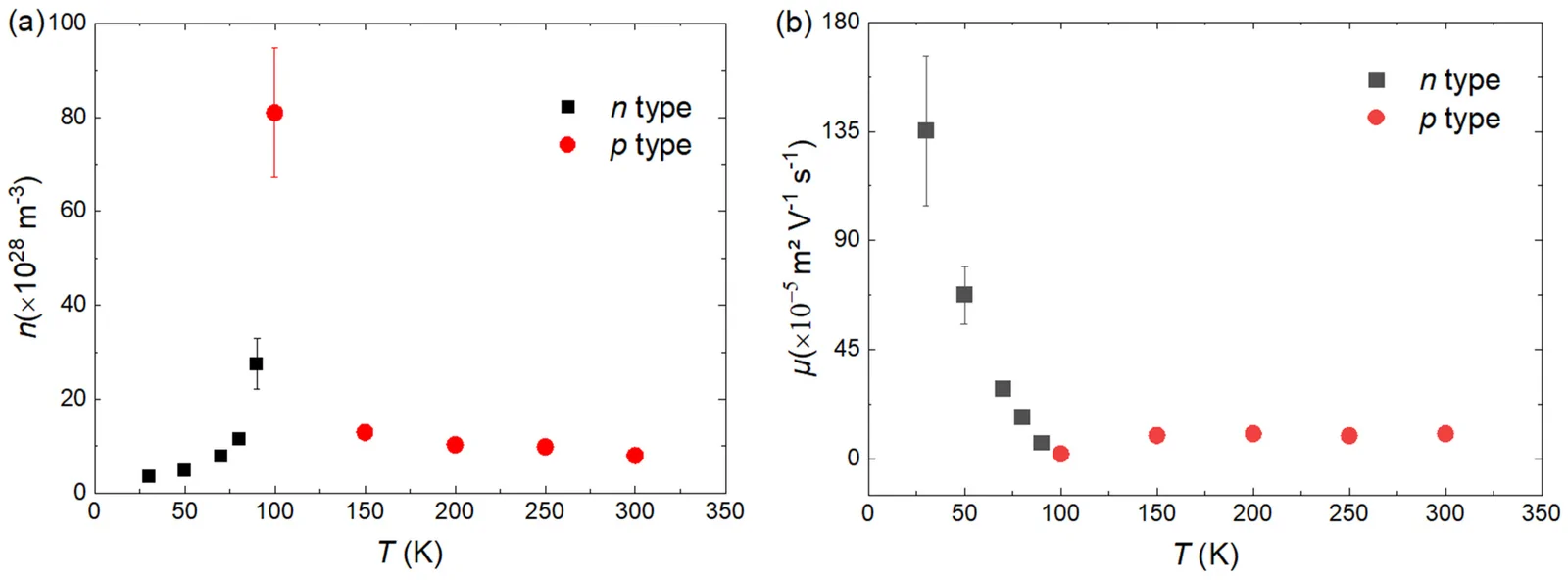
(**a**) Temperature-dependent apparent carrier density *n*_app_(*T*) extracted from the high-field Hall resistivity using the single-band approximation *n*_app_ = 1/(*e*|*R_H_*|), where *R_H_* = *dρ_xy_*/*dH*. Black squares and red circles denote *n*-type and *p*-type transport, respectively. Error bars represent the standard deviation propagated from the linear fits of *ρ_xy_*(*H*) in the high-field region (5–9 T). (**b**) Temperature-dependent apparent mobility *μ*_app_(*T*) obtained from *μ*_app_ =|*R_H_*|/*ρ_xx_*. Error bars are calculated from the standard error of the linear fits and the uncertainty in *ρ_xx_*.

**Figure 5:**
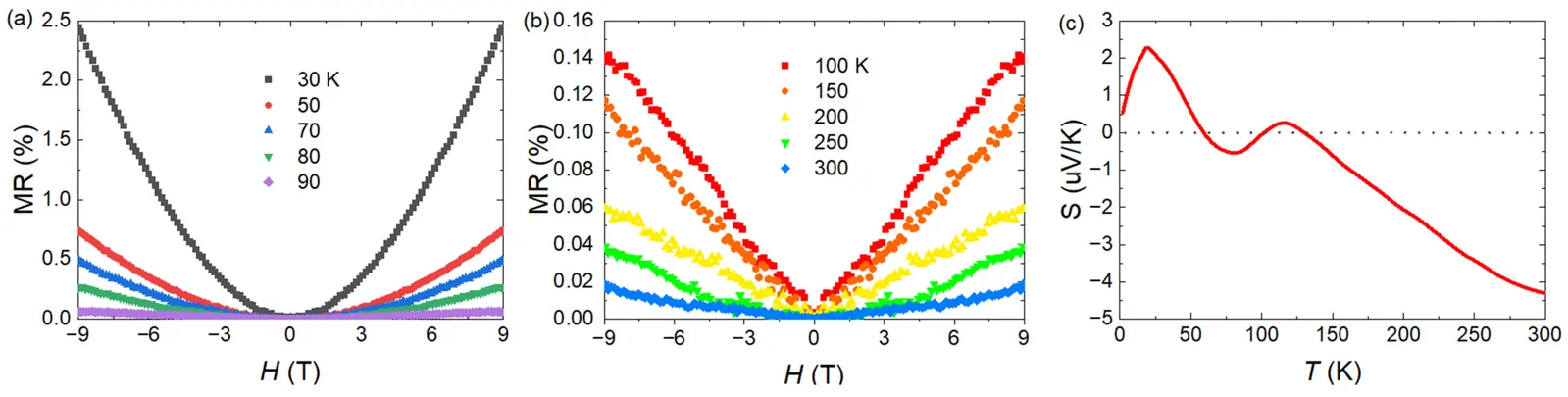
(**a**) Magnetoresistance at temperatures of 30, 50, 70, 80, and 90 K. (**b**) Magnetoresistance at temperatures of 100, 150, 200, 250, and 300 K. (**c**) Temperature-dependent thermopower *S*(*T*).

The apparent carrier density *n*_app_(*T*) and mobility *μ*_app_(*T*) are shown in Figure 4a,b. These values were extracted using the single-band approximation described in the Experimental Methods Section. Because the system is multiband, these values should be regarded as effective single-band parameters rather than absolute carrier concentrations of individual bands. Above *T_CDW_*, *n*_app_ is positive and corresponds to hole-dominated transport with low mobility. Below *T*_CDW_, the Hall sign reverses and *n*_app_ becomes negative, indicating electron-dominated transport. This sign reversal is consistent with CDW-driven Fermi-surface reconstruction. The error bars in Figure 4a,b represent the standard deviation propagated from the linear fits of *ρ_xy_*(*H*). As shown in Figure 5a,b, the MR curves form symmetric parabolas over the entire temperature range, with the MR amplitude increasing substantially below *T_CDW_*. This enhancement is attributed to the CDW gap, which suppresses interband scattering and enhances the single-band contribution, thereby further supporting the picture of CDW-induced Fermi-surface reconstruction. The thermopower *S*(*T*) in Figure 5c changes sign from positive at low temperatures to negative above ~100 K, consistent with the coexistence of electron- and hole-type carriers and a multiband Fermi surface [18]. This behavior is qualitatively consistent with the carrier-type reversal observed in the Hall measurements, although the sign of *S* is also influenced by scattering mechanisms [19].

**Figure 6:**
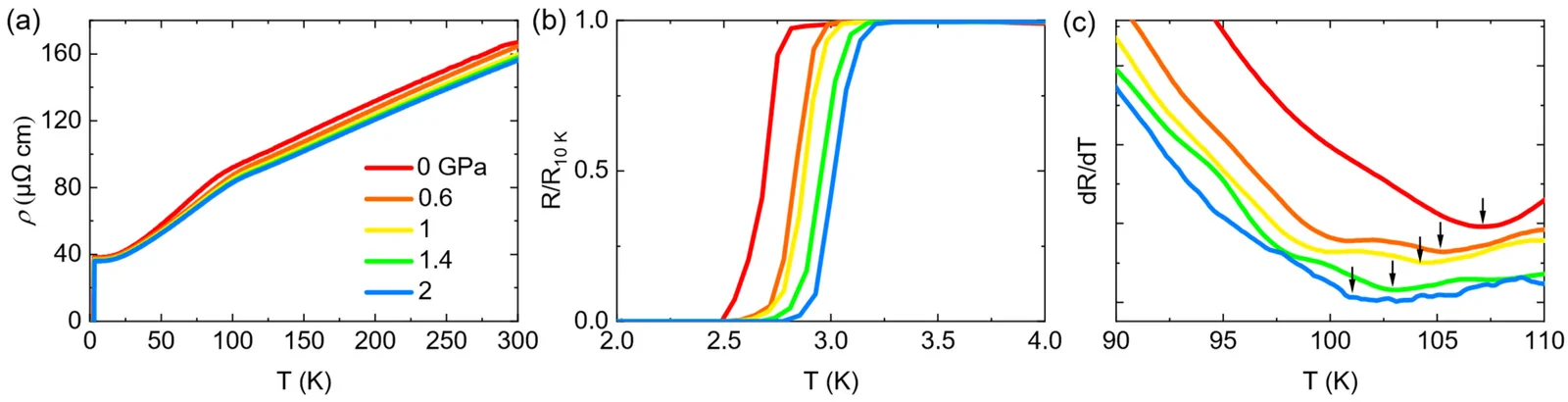
(**a**) Temperature dependence of resistivity *ρ*(*T*) under various pressures up to 2 GPa. The downward shift in *ρ*(*T*) with increasing pressure is observed mainly in the normal-state region above *T_CDW_*. (**b**) Normalized superconducting transition within an enlarged region at a low temperature at different pressures, measured at zero magnetic field. (**c**) d*R*/d*T* curves as a function of temperature at different pressures. The arrows indicate the CDW phase transition (black).

The temperature-dependent resistivity under various pressures is shown in Figure 6a. The minimum of the *dR*/*dT* curves corresponds to the CDW transition temperature: as pressure increases from 0 to 2 GPa, *T_CDW_* monotonically decreases from 107 K to 100 K, exhibiting an approximately linear pressure dependence. The physical origin lies in hydrostatic compression of the interlayer spacing, which weakens the in-plane Ta-Se orbital hybridization and reduces Fermi-surface nesting, thereby suppressing the CDW lattice distortion [14]. In the normal-state region (i.e., above *T_CDW_* and outside the superconducting transition), the resistivity *ρ*(*T*) curves shift downward with increasing pressure. At a given temperature in this region, higher pressure yields lower resistivity, indicating enhanced orbital hybridization and improved carrier mobility. Near the CDW and superconducting transitions, the pressure dependence is more complex: the CDW-related resistance step weakens with pressure, while the superconducting transition shifts to higher temperature, as discussed below. The low-temperature normalized superconducting resistance *R*/*R*_10K_ curves are shown in Figure 6b. They show that with increasing pressure, the superconducting transition window shifts continuously to higher temperatures and *T_c_* rises monotonically, in direct opposition to the decreasing *T_CDW,_* clear evidence of the mutual competition between CDW and superconductivity. Field-dependent superconducting resistance transitions were measured for two field orientations in Figure 2. *H*⊥*ab* (field perpendicular to layers): Superconductivity is destroyed at low fields, with low *H_c2_* values. *H*∥*ab* (field parallel to layers): Superconductivity can withstand fields up to 5 T, with *H*_c2_ much larger than the perpendicular case, consistent with classical 2D superconductors such as NbSe_2_ [12] and TaS_2_ [13]. The *H_c_*_2_(*T*) data were fitted using the anisotropic Ginzburg–Landau model. The fits yield $H_{c2}^{⊥\mathrm{ab}}$(0) = 2.6 T and $H_{c2}^{∥\mathrm{ab}}$(0) = 12.5 T, giving an anisotropy ratio *γ* = $H_{c2}^{∥\mathrm{ab}}$(0)/$H_{c2}^{∥\mathrm{ab}}$(0) ≈ 4.81. The corresponding coherence lengths are *ξ_ab_* ≈ 11.3 nm and *ξ_c_* ≈ 2.34 nm. These values confirm the quasi-two-dimensional superconducting behavior of bulk 3R-TaSe_2_. It should be noted that the lower critical field *H_c_*_1_ was not measured in this work due to the limitation of the high-pressure cell, which does not allow magnetization measurements. The present study focuses on *H_c_*_2_ to reveal the quasi-two-dimensional superconducting nature.

**Figure 7:**
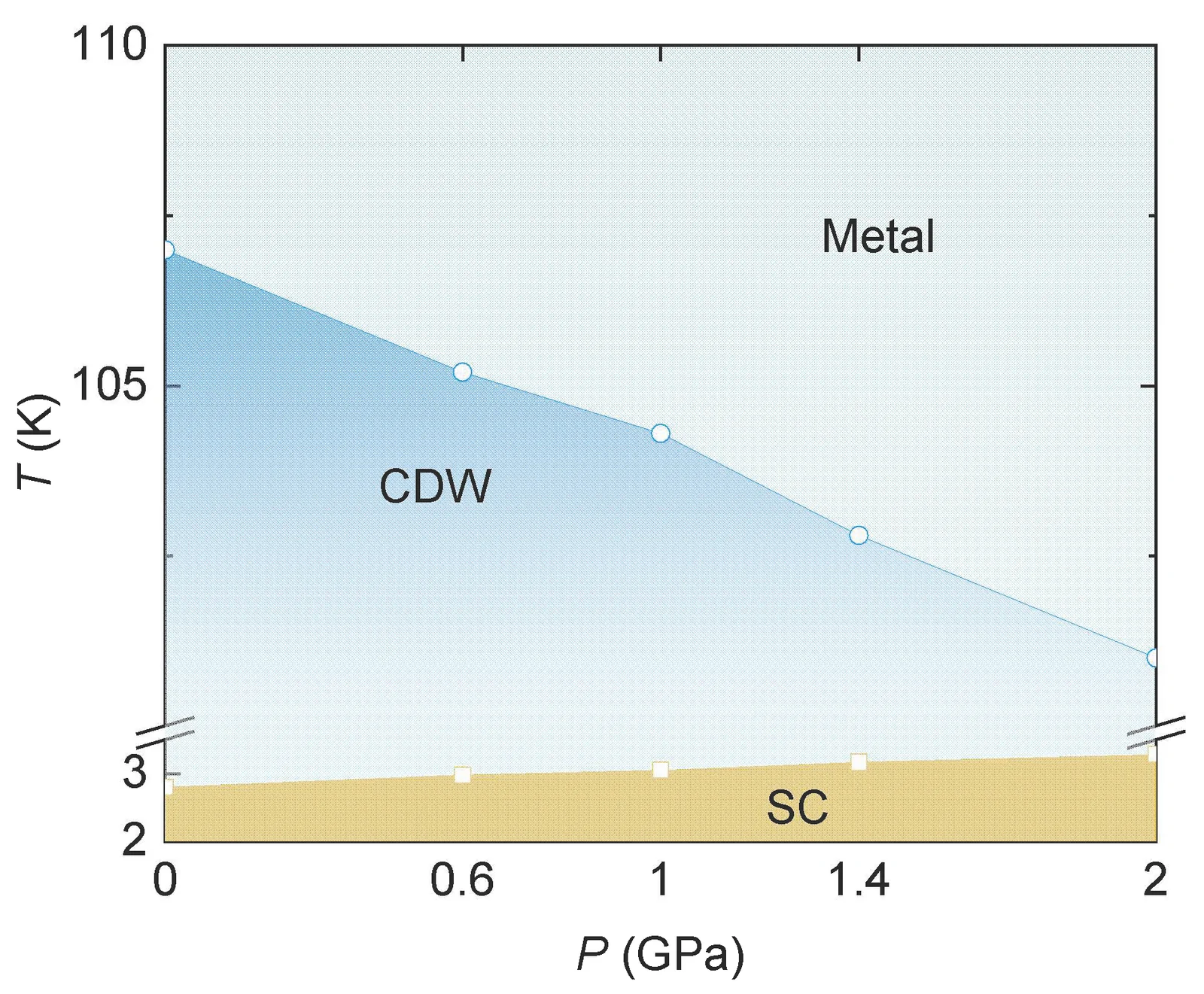
The phase diagram of 3R-TaSe_2_. Hollow circles represent *T_CDW_* from the electrical resistivity measurements, and the hollow squares correspond to *T*_c_. Both are extracted from zero-field resistivity measurements.

As shown in Figure 7, we summarized *T_CDW_* and *T*_c_ as a function of pressure from resistivity measurements. The light-blue region represents the CDW-ordered metallic phase, the light-green region is the normal metallic phase, and the bottom yellow narrow band denotes the superconducting phase. The CDW phase boundary slopes downward monotonically with pressure, while the superconducting region gradually widens with increasing *T_c_*, clearly illustrating the competition relationship between the two quantum phases and providing quantitative constraints for theoretical analysis. The phase diagram in Figure 7 is based on zero-field measurements. Since the CDW transition is only weakly sensitive to magnetic field, while *T_c_* is strongly suppressed by the field, the zero-field phase diagram is appropriate for revealing the intrinsic CDW–SC competition. A magnetic-field-dependent phase diagram would be an interesting extension for future work [20].

## 4. Discussion

CDW and superconductivity share the same Fermi-surface electrons, giving rise to a natural competition [21]: the CDW transition opens a gap via Fermi-surface nesting, consuming Fermi-surface density of states *N*(*E_F_*). In BCS theory, *T_c_* is positively correlated with *N*(*E_F_*), so the loss of density of states directly suppresses Cooper-pair formation, resulting in a low ambient-pressure *T_c_* [22]. Upon applying hydrostatic pressure, the interlayer spacing is reduced, which weakens the in-plane Ta-Se distortion, mismatches the nesting vector, and progressively closes the CDW gap. Consequently, *N*(*E_F_*) is restored and electron–phonon coupling is enhanced, leading to an increase in *T_c_* [4]. In this pressure range, within the 0–2 GPa range, no other ordered phases (e.g., spin-density wave or Mott insulator) emerge; the phase evolution is dominated solely by CDW-superconductivity competition, without additional degrees of freedom, making this an ideal two-order-parameter competition model for theoretical studies.

Compared with the widely studied 2H-TaSe_2_ [18,23], the 3R phase features a triple-layer stacking that lowers crystal symmetry and splits the Fermi surface into more electron and hole pockets, resulting in the double sign reversal observed in both Hall and thermopower, a multiband transport behavior [14]. In the 2H phase, Fermi-surface nesting gives rise to only a single dominant carrier type, lacking this characteristic. this serves as a key transport identifier distinguishing the two polytypes. The strong anisotropy of superconductivity confirms that bulk 3R-TaSe_2_ is a quasi-two-dimensional superconductor [10]: interlayer van der Waals coupling is much weaker than intralayer covalent hybridization, so the superconducting coherence length along the *c*-axis is extremely short [14]. A perpendicular magnetic field easily destroys Cooper pairs, whereas an in-plane field only affects the intralayer supercurrent, requiring a much higher critical field-consistent with classic 2D superconductors such as NbSe_2_ [12] and TaS_2_ [13].

Future efforts could extend pressure measurements to higher regimes, exceeding 2 GPa, in order to determine whether a superconducting dome emerges following the suppression of the CDW phase and whether a quantum critical point exists. These measurements would be complemented by high-pressure angle-resolved photoemission spectroscopy (ARPES) to directly observe the pressure-induced closure of the charge gap and the evolution of the Fermi surface, thereby verifying the reconstruction scenario inferred from transport experiments [19]. In addition, ionic intercalation with species such as Li^+^ and K^+^ will be performed to independently tune the interlayer spacing and carrier concentration. A systematic comparison between chemical intercalation and hydrostatic pressure will help disentangle the effects of lattice compression from those of charge doping [24]. Furthermore, ultrafast spectroscopy and nuclear magnetic resonance could be employed to probe collective excitations and spin dynamics in the CDW state, providing deeper insight into the microscopic coupling between the CDW and superconductivity.

## 5. Conclusions

In summary, we have grown a 3R-TaSe_2_ single crystal by the chemical vapor transport method. The CDW transition emerges at ~100 K, and bulk superconducting transition appears below ~2.8 K under ambient pressure. Hall effect and thermopower measurements jointly demonstrate that the CDW transition induces Fermi-surface reconstruction, with coexisting electron and hole pockets and a carrier-type reversal across *T_CDW_*. Hydrostatic pressure monotonically suppresses CDW order while simultaneously enhancing superconductivity, establishing clear antagonistic competition. The pressure-induced interlayer compression weakens lattice distortion and closes the CDW gap, thereby restoring the Fermi-surface density of states and strengthening electron–phonon coupling. Further, the large difference between in-plane and out-of-plane upper critical fields confirms that bulk 3R-TaSe_2_ is a quasi-two-dimensional superconductor, with weak interlayer van der Waals coupling as the origin of anisotropy. Based on all pressure-dependent data, a *P*(*T*) phase diagram is constructed, quantitatively illustrating the mutual suppression and evolution of the CDW and superconducting phases. This work clearly reveals the competitive physical picture between CDW and superconductivity in undoped 3R-TaSe_2_, providing a clean and comprehensive experimental benchmark for understanding correlated electronic order competition in layered van der Waals materials, and offering experimental support and theoretical guidance for the pressure-engineering of correlated electronic states and design of novel low-dimensional superconducting systems.

## Data Availability

The original contributions presented in this study are included in the article. Further inquiries can be directed to the corresponding authors.
